# The impact of preventive behaviors on self-rated health, depression symptoms, and daily functioning among middle-aged and elderly Chinese: An empirical study

**DOI:** 10.1371/journal.pone.0305672

**Published:** 2024-07-16

**Authors:** Yuehong Zhang, Wenbin Zang, Manxia Tian, Yumiao Zhang

**Affiliations:** 1 School of Public Administration, Southwestern University of Finance and Economics, Chengdu, Sichuan, China; 2 Hebei Aademy of Social Sciences, Shijiazhuang, Hebei, China; University of Jyvaskyla, FINLAND

## Abstract

**Introduction:**

With the intensifying issue of an aging population, the health of middle-aged and elderly individuals garners increased attention. Preventive behaviors are pivotal in enhancing life quality and extending healthy living. This study examines the effects of preventive behaviors on self-rated health, depression, and daily functioning among these populations.

**Materials and methods:**

Drawing on data from the China Health and Retirement Longitudinal Study (CHARLS), this research applies a panel ordered probability model to scrutinize the influence of preventive behaviors on health outcomes among middle-aged and elderly populations. It utilizes self-rated health, depression, and daily functioning as pivotal health indicators to assess the effects.

**Results:**

Preventive behaviors exert a significant impact on self-assessed health and daily functioning among middle-aged and elderly populations. Engagement in social activities effectively reduces depression symptoms. Primary preventive measures, including physical and social activities, enhance health outcomes through medical consultations. Conversely, secondary preventive actions, such as undergoing physical examinations, facilitate early detection of diseases, enabling timely intervention and health advisories. It is noteworthy that individuals with higher incomes derive lesser benefits from these physical or social endeavors.

**Conclusion:**

Sociodemographic determinants such as age, income, and educational attainment significantly modulate the efficacy of preventive behaviors on the health outcomes of middle-aged and elderly populations. This research underscores the pivotal role of physical examination services within primary healthcare frameworks and advocates for the tailoring of health promotion strategies to the accessible social needs and engagements of economically and educationally disadvantaged seniors.

## 1.Introduction

Health is a fundamental human necessity and a key objective in human development, widely recognized as an essential human capability and a fundamental freedom [1]. The state of an individual’s health not only impacts personal and familial well-being but also influences the efficiency of socioeconomic dynamics. The deepening extent of population aging has propelled geriatric health issues into the spotlight. As of the end of 2021, China’s elderly population aged 60 and above reached 267 million, constituting 18.9% of the total population; the population aged 65 and above surpassed 200 million, accounting for 14.2%. It is projected that during the "Fourteenth Five-Year Plan" period, the elderly population over 60 will exceed 300 million, exceeding 20% of the total demographic. By 2035, the number of seniors over 60 is expected to rise to approximately 420 million, surpassing 30% of the overall population. As the aging process intensifies, China is progressively entering an era of advanced aging. Consequently, addressing the health concerns of the middle-aged and elderly, who are more susceptible to diseases, has emerged as a pressing social issue [2].

Chronic conditions, mental depression, and restricted mobility have emerged as significant determinants affecting the health and life quality of the elderly [3]. The presence of multiple chronic diseases can trigger negative emotional responses, thereby continuously elevating the incidence of depression among the elderly population [4,5]. The China Elderly Health Research Report (2020–2021) highlights that chronic diseases are the predominant health concern among the elderly. Statistics reveal that among Chinese citizens aged 60 and above, the prevalence rates of hypertension, diabetes, and hypercholesterolemia are 58.3%, 19.4%, and 10.5%, respectively. Furthermore, over three-quarters of this demographic suffer from multiple co-morbidities, with a noticeable increase in chronic disease incidence correlating with advancing age.The socio-demographic shifts, including declining birth rates and an increase in elderly widowhood, have resulted in a growing number of elderly individuals living alone or residing in care facilities. Consequently, mental health issues among this demographic, particularly depression, warrant increased attention. The China National Mental Health Development Report indicates that depression is a prevalent psychological condition among the elderly. Chronic depression not only predisposes to clinical depression but may also escalate into severe manifestations like self-harm [6]. Moreover, depression in the elderly often intertwines with other medical conditions, exacerbating physical ailments and leading to irreversible cognitive decline, potentially culminating in dementia [7]. With the natural decline in physical functions, the elderly’s capacity for daily activities, such as eating and dressing, becomes increasingly compromised. According to the National Health Commission’s statistics, in 2021, over 190 million elderly individuals in China were afflicted with chronic illnesses, with approximately 40 million experiencing disability or semi-disability. Addressing the decline in daily activity capabilities among the elderly necessitates a dual approach: firstly, enhancing the overall health status of the elderly to promote healthy aging and delay the onset of disability, and secondly, establishing a comprehensive caregiving system to support disabled elderly individuals.

In the realm of enhancing geriatric health, traditional research primarily centered on advancements in medical technology, provision of healthcare services, and the role of familial caregiving. Recent scholarly discourse, however, has shifted to underscore the significance of home-based and institutional care approaches. These methods are increasingly recognized not only for offering diverse care options to the elderly but also for their vital contribution to improving physical and mental well-being and managing chronic illnesses, as highlighted in recent studies [8–10]. Furthermore, Hua Ying’s (2017) research underscores that disease prevention and proactive health management are pivotal in curbing the progression of chronic conditions [11]. Of particular interest is the growing recognition of individual behavior’s impact on preventing disease onset and mitigating health deterioration, a topic garnering widespread attention in medicine and health economics [12–15]. Despite this global focus, Chinese academic research predominantly fixates on factors like gender, age, chronic diseases [16–18], socioeconomic traits [19–21], and external determinants such as living conditions, healthcare infrastructure, or policy impacts on health disparities [22–26]. Nevertheless, the influence of individual behaviors on geriatric health remains underexplored within Chinese scholarly circles. This gap necessitates a more profound exploration and analysis, aiming to thoroughly comprehend and evaluate the latent role of individual behaviors in sustaining and elevating elderly health.

This study delves into the pivotal role of individual behaviors in enhancing geriatric health, particularly through the lens of preventive medicine, thereby addressing a notable research void in China’s health economics arena. In the domain of preventive medicine, individual behaviors are categorically differentiated into primary, secondary, and tertiary prevention, contingent upon their effectiveness at various disease stages [27,28]. This delineation serves as the cornerstone for the classification of preventive behaviors in this analysis. The research categorizes individual actions into primary preventive measures such as diet, exercise, and social interaction, and secondary preventive measures including routine health check-ups. The aim is to scrutinize their distinct impacts on the health of the elderly. Leveraging comprehensive individual data from the China Large-scale Microdata Survey Library (CHARLS) coupled with robust economic research methodologies, this study conducts an empirical examination of the profound linkages between preventive behaviors and key health indicators: self-assessed health, mental depression, and daily functional capabilities. Additionally, by stratifying the data by age and income, the study unveils nuanced insights into the influence of preventive behaviors on geriatric health across diverse socioeconomic strata. In evaluating geriatric health, this research adopts three critical dimensions due to their encompassing nature and predictive validity. Self-rated health, indicative of an individual’s subjective perception of their health status, emerges as a vital predictor of long-term health outcomes [29]. Mental depression, a direct determinant of psychological well-being and overall health [30], significantly impacts elderly health. Furthermore, daily functioning offers tangible insights into the self-care and caregiving needs of the elderly. The synergistic application of these dimensions underscores the comprehensive and pragmatic approach of the study. In essence, this paper substantially contributes to the discourse on individual preventive behaviors in geriatric health maintenance and enhancement, particularly crucial in an era where the focus predominantly skews towards disease treatment, often overshadowing the importance of preventive actions.

## 2.Literature review

The body of research literature extensively explores the intricate relationship between individual behavior and health outcomes. Pioneering this field, Grossman’s (1972) study laid a foundational framework by proposing a health input-output model, highlighting the crucial role of investing in health capital [12]. This includes elements such as medical care, diet, and exercise, thereby offering a valuable lens to comprehend how individual health behaviors contribute to building health capital. With the progression of research, the spectrum of behavioral factors under consideration has broadened, encompassing a diverse array of dimensions. These range from diet and physical activity to social engagement, and extend to behaviors like smoking, alcohol consumption, substance use, and adherence to regular health screenings. The connections between these varied behaviors and resultant health outcomes have been increasingly elaborated upon and empirically validated in a series of subsequent studies.

Dietary habits exhibit a strong correlation with disease morbidity and mortality rates, with poor dietary patterns contributing to deteriorating physical and mental health among the elderly [31–33]. Research indicates that maintaining wholesome dietary practices, coupled with a healthy lifestyle encompassing leisure and social activities, significantly enhances health levels and reduces depression risks [34–36]. Engaging in physical activity is known to substantially lower the risk of various diseases, though the incremental health benefits of increased activity intensity tend to diminish [37]. Moreover, individuals with higher physical activity levels are less prone to depression [38,39]. Active older adults not only face reduced risks of all-cause mortality and diverse health complications but also enjoy better life quality and cognitive functions [40]. Studies on the behavior of undergoing physical examinations confirm their positive impact on the elderly’s survival rates [41], and significantly contribute to improving self-assessed health statuses among rural elderly populations [42]. Furthermore, regular health screenings play a pivotal role in enhancing chronic disease diagnosis and in reducing overall disease risks and mortality [43,44]. However, the effect of regular health check-ups on health improvement appears to be relatively limited for the middle-aged demographic [45].

Emerging research indicates that smoking adversely impacts a range of bodily functions in the elderly and is linked to an increased likelihood of depression onset [13,46]. Additionally, smoking is implicated in causing cancers, deteriorating oral health, and hastening the process of immune system aging [47,48]. The influence of alcohol consumption on older adults’ health is multifaceted, influenced by variables such as the quantity, duration, type, frequency of consumption, and individual factors [49]. While moderate alcohol intake may offer benefits in terms of stress reduction, social and cognitive engagement, and lowering the risk of certain diseases [50–52], excessive drinking can lead to an array of health issues, including cardiovascular diseases, cancer, cognitive impairments, and elevated mortality rates [53,54]. Furthermore, sedentary behavior emerges as a significant health risk for the elderly, particularly in reducing the basal metabolic rate and energy expenditure [55]. Studies have established a correlation between prolonged sedentary lifestyle and increased rates of morbidity, mortality, obesity, metabolic syndrome, mental health challenges, and dementia among older adults [56–58]. This is especially pronounced in the female demographic, where sedentary behavior has been significantly linked to reduced physical function and a heightened risk of cardiovascular diseases, with older women being particularly susceptible [59–61].

In synthesizing the current literature on the nexus between individual behaviors and health outcomes, several critical gaps warrant attention. Firstly, while existing studies have probed into various health dimensions such as self-assessed health, depression, and mortality, a comprehensive scrutiny of the causal links between individual behaviors and the overall health status of the elderly remains elusive. Secondly, despite the recognition of correlations between individual behaviors and health outcomes, detailed analyses of these pathways are yet to be thoroughly explored. Lastly, the current economic research on individual behavior and health somewhat lacks in-depth understanding of the intricacies of individual behaviors, especially in terms of their differential roles across stages of disease prevention. Addressing these limitations, this study aims to unravel the complex relationship between individual behavior and elderly health from the vantage point of preventive actions. By integrating economic methodologies with in-depth analyses of extensive micro-survey data, the research strives to not only enhance the comprehension of the individual behavior-health interplay but also to furnish a more nuanced and empirical examination. Such an approach is poised to significantly contribute to theoretical and practical insights, offering a solid foundation for the formulation of future health interventions and policy initiatives.

## 3. Materials & methods

### 3.1 Materials

The data in this paper come from the 2011, 2013, and 2015 China Pension and Tracking Survey (CHARLS), which constructs unbalanced panel data with a total of 56,033 sample individuals. (CHARLS) collects high-quality information on individuals and their households of middle-aged and elderly people aged 45 years and older in China, covering 150 counties and 450 communities (villages) in 28 provinces (districts and municipalities) across the country. The baseline and follow-up questionnaires mainly include basic personal information, family structure, health status, health service utilization, work, income, and basic community information.

### 3.2 Description of the variable

In this paper, we referenced Zhou Qin (2018), Hong Haoqi et al. (2021), and Li Yaqing et al. (2022) for indicator selection for self-assessed health, depression, and ADL ability [62–64]. The self-rated health variable derives from CHARLS questionnaire responses ranging from ’very good’ to ’very bad,’ assigned values 1 to 5, respectively. Depression symptoms was assessed using the CES-D scale from the questionnaire, based on Andreson et al. (1994) [65], covering ten dimensions of mental state. This scale includes both negative and positive mood indicators, with responses ranging from ’rarely’ to ’most of the time,’ scored between 1 and 4. The total CES-D score, ranging from 0 to 40, indicates the level of depression. Daily functioning was measured using a combination of ADL and IADL impairments from CHARLS, where ADL encompasses six self-care activities and IADL includes five household tasks. A score of 1 represents a need for assistance, indicating a more severe physical health condition.

In this paper, the definition of preventive behaviors refers to actions and measures taken by individuals or groups aimed at reducing disease incidence, decreasing health risks, and associated medical costs. These behaviors not only include personal health practices such as maintaining a healthy diet and regular exercise but also public health interventions. The core explanatory variables were primary preventive behaviors (physical and social activities) and secondary preventive behavior (physical examination). Physical activity was determined by CHARLS questionnaire responses to high, moderate, or light-intensity activities, with any affirmative answer scored as 1. Social activity was based on participation in any listed CHARLS social activities, scored as 1 for participation and 0 for non-participation. Physical examination frequency was derived from the most recent routine check-up data, scored as 1 if within the last two years.

Control variables included age, gender, education years, marital status, urban/rural residence, last year’s total income, and health insurance enrollment. Mediating variables were hospitalization, smoking, and alcohol consumption, used to examine the influence mechanism on health among middle-aged and elderly

### 3.3 Methods

In this study, we employ self-assessed health status, mental depression symptoms, and daily functioning as dependent variables, while primary preventive behaviors encompassing physical and social activities, as well as secondary preventive behaviors such as physical examinations, are utilized as core independent variables. To comprehensively evaluate the influence of these preventive behaviors on the health outcomes of middle-aged and elderly individuals, a panel ordered multinomial choice Probit regression model is constructed as the baseline model. This model integrates various control variables to provide a robust analysis of the determinants impacting the health of this demographic group. The structure of the model is delineated as follows:

$$\ln\left[\frac{P(H_{i}<j)}{1-P(H_{i}<j)}\right]=\alpha_{0}+\alpha_{1}{ph\_behavior}_{ijt}+\alpha_{2}{so\_behavior}_{ijt}+\alpha_{3}{ex\_behavior}_{ijt}+{Control}^{\prime }\alpha_{k}+\varepsilon_{ijt}$$ (1)

(1)i and t represent the individual number and survey year of the rural middle-aged and elderly individuals, respectively; j represents the option of choosing self-assessed health, and takes the values of 1, 2, 3, 4, and 5; *ph_behavior*_*ijt*_represents whether to participate in physical activities or not, *so_behavior*_*ijt*_ represents whether to participate in social activities or not, *α*_3_*ex_behavior*_*ijt*_ represents whether to participate in the physical examination programme and *Control*′ represents a series of control variables.

### 3.4 Ethical considerations and data source

The data for this study comes from the China Health and Retirement Longitudinal Study (CHARLS), a publicly available database provided by Peking University. Ethical approval for all CHARLS waves was granted by the Institutional Review Board at Peking University, with the approval number: IRB00001052-11015. The CHARLS database is an openly accessible micro-database in China, with resources made publicly available. Access to the data can be found at the following link: https://charls.pku.edu.cn/.

## 4. Analysis of empirical results

### 4.1 Descriptive statistics

Descriptive statistics of the variables were conducted prior to regression analysis, with the results presented in Table 1.

**Table 1:** Descriptive statistics.

Variable	Define	mean	Std.Error
**Self-assessed Health**	1 = very good, 2 = good, 3 = fair, 4 = bad, 5 = very bad	3.4715	1.0381
**Depression symptoms**	Values range from 0 to 40, with higher values indicating more severe depression	17.3731	6.6859
**Daily Functioning**	0 = no difficulty, 1 = difficulty	0.3985	0.4896
**Physical Activity**	0 = not involved, 1 = involved	0.8931	0.3090
**Social Activity**	0 = not involved, 1 = involved	0.5542	0.4971
**Physical Examination**	0 = not involved, 1 = involved	0.4856	0.4998
**Healthcare Utilization**	0 = not hospitalized, 1 = hospitalized	0.1170	0.3214
**smoking**	0 = no, 1 = yes	0.1844	0.3878
**drinking**	0 = no, 1 = yes	0.3419	0.4744
**Sex**	0 = female, 1 = male	0.4830	0.4997
**Age**	Actual age	60.4259	9.9705
**Edu**	1 = illiterate, 6 = elementary and below, 9 = middle school. 12 = High school, 16 = University and above	6.1256	3.8132
**Urban**	0 = rural, 1 = urban	0.4037	0.4906
**Married**	0 = not in marriage, 1 = in marriage	0.8687	0.3377
**Medical insurance**	0 = no, 1 = yes	0.6028	0.4893
**Income**	Income is logarithmic	7.9440	2.1421

### 4.2 Benchmark regression analysis

Table 2 reports regression results for the core explanatory variables in columns (1), (3), (5), while columns (2), (4), and (6) present the results after adding control variables, with all regressions accounting for province and year. The findings, presented as coefficients and t-values, indicate that physical and social activities, as well as physical examination behaviors, significantly influence self-rated health, depression symptoms, and daily functioning. These effects remain significant after including control variables. Notably, the influence of physical activity on depression symptoms becomes insignificant with the addition of control variables, suggesting that other factors may have a more substantial impact on depression symptoms than physical activity.

**Table 2:** Results of basic regression analysis.

	Self-assessed Health		Depression symptoms		Daily Functioning	
	(1)	(2)	(3)	(4)	(5)	(6)
**Physical Activity**	-0.194***	-0.133***	-0.115***	-0.051	-0.426***	-0.314***
	(-6.45)	(-4.04)	(-4.16)	(-1.67)	(-11.42)	(-7.36)
**Social Activity**	-0.123***	-0.0593**	-0.165***	-0.122***	-0.241***	-0.169***
	(-7.16)	(-3.15)	(-10.01)	(-6.73)	(-9.71)	(-6.06)
**Physical Examination**	0.0840***	0.111***	0.0204	0.0489**	0.106***	0.123***
	(4.81)	(5.79)	(1.23)	(2.69)	(4.160	(4.29)
**Sex**		-0.143***		-0.226***		0.0185
		(-7.35)		(-12.29)		(0.62)
**Age**		0.0083***		0.0027**		0.0179***
		(7.63)		(2.69)		(11.29)
**Edu**		-0.0101***		-0.0167***		-0.0322***
		(-3.40)		(-5.84)		(-7.01)
**Urban**		-0.119***		-0.0984***		-0.111***
		(-5.68)		(-4.98)		(-3.52)
**Married**		0.0521		-0.185***		-0.0263
		(1.77)		(-6.57)		(-0.65)
**Medical insurance**		0.101		-0.0533		-0.0954
		(1.86)		(-1.09)		(-1.24)
**Income**		-0.0663***		-0.0728***		-0.0547***
		(-13.67)		(-15.68)		(-7.36)
**Regional Fixed**	**YES**	**YES**	**YES**	**YES**	**YES**	**YES**
**Year Fixed**	**YES**	**YES**	**YES**	**YES**	**YES**	**YES**
**Observations**	16160	14091	16159	14031	11348	9654
**Wald chi2**	491.77	1066.71	731.23	1571.24	477.21	773.95
**Pseudo R2**	0.011	0.0259	0.0073	0.0183	0.0328	0.0675

Note: In the table, values in brackets represent robust standard errors

*, **, and *** indicate statistical significance at the 10%, 5%, and 1% levels, respectively. Subsequent references will not be repeated."

## 5. Further analysis

### 5.1 Regression results of impact mechanisms

A mechanistic analysis reveals how preventive behaviors impact self-assessed health, depression symptoms, and daily competence in middle-aged and older adults. Participation in physical activities reduces illness incidence, leading to fewer medical visits and hospitalizations, thereby improving the health of this demographic. Table 3 (1) shows regression results for the impact of physical activity on healthcare-seeking behavior, while (2), (3), and (4) detail its effects on self-assessed health, depression symptoms, and daily functioning. Table 4 (1) presents the influence of social activities on healthcare access, with (2), (3), and (4) demonstrating their effects on the same health outcomes. These results confirm the significant role of physical and social activities in influencing health.

**Table 3:** Regression results for the mediating effect of physical activity.

	Healthcare	Self-assessed Health	Depression	Daily Functioning
	(1)	(2)	(3)	(4)
**Physical Activity**	-0.194***	-0.118***	0.306***	-0.320***
	(-4.56)	(-3.79)	(11.27)	(-7.94)
**Healthcare**		0.539***	-0.0610*	0.273***
		(18.81)	(-2.10)	(7.35)
**Controls**	**YES**	**YES**	**YES**	**YES**
**Regional Fixed**	**YES**	**YES**	**YES**	**YES**
**Year Fixed**	**YES**	**YES**	**YES**	**YES**
**Observations**	15546	15531	15466	10620
**Wald chi2**	390.18	1477.46	1725.45	855.91
**Pseudo R2**	0.0372	0.0337	0.0186	0.0677

**Table 4:** Regression results for the mediating effect of socialization.

	Healthcare	Self-assessed Health	Depression	Daily Functioning
	(1)	(2)	(3)	(4)
**Social Activity**	-0.0444*	-0.0744***	-0.129***	0.279***
	(-2.56)	(-6.74)	(-12.18)	(12.3)
**Healthcare**		0.543***	0.286***	-0.132***
		(30.58)	(17.39)	(-8.19)
**Controls**	**YES**	**YES**	**YES**	**YES**
**Regional Fixed**	**YES**	**YES**	**YES**	**YES**
**Year Fixed**	**YES**	**YES**	**YES**	**YES**
**Observations**	40289	40241	40072	27661
**Wald chi2**	921.14	3585.38	4260.2	2146.67
**Pseudo R2**	0.0335	0.0318	0.0178	0.0627

Table 5 (1) shows the impact of physical examinations on healthcare-seeking behavior, with (2), (3), and (4) exploring their influence on self-assessed health, depression symptoms, and daily functioning, all adjusted for provincial and yearly effects. Table 6 (1) and (2) present the effects of physical examinations on smoking and drinking behaviors, while (3) and (4) show their impact on self-rated health with the inclusion of these variables. The findings indicate that physical examination behaviors significantly affect the self-rated health of middle-aged and older adults by altering their smoking and drinking behaviors.

**Table 5:** Regression results for the mediating effect of Physical examination.

	Healthcare	Self-assessed Health		Depression	Daily Functioning
	(1)	(2)		(3)	(4)
**Physical examination**	0.355***	0.0359**	-0.00239		0.0665***
	(19.22)	(3.07)	(-0.21)		(3.82)
**Healthcare**		0.548***	0.257***		0.284***
		(30.31)	(14.94)		(12.26)
**Controls**	**YES**	**YES**	**YES**		**YES**
**Regional Fixed**	**YES**	**YES**	**YES**		**YES**
**Year Fixed**	**YES**	**YES**	**YES**		**YES**
**Observations**	38094	37995	37257		26009
**Wald chi2**	1217.41	3438.58	3874.31		2116
**Pseudo R2**	0.0452	0.0323	0.0173		0.0657

**Table 6:** Regression results for the mediating effect of Physical examination.

	smoking	drinking	Self-assessed Health	Self-assessed Health
	(1)	(2	(3)	(4)
**Physical examination**	-0.0977***	-0.0600***	0.0777***	0.0678***
	(-4.30)	(-3.89)	(5.93)	(5.83)
**smoking**			-0.0492*	
			(-2.55)	
**drinking**				-0.190***
				(-14.76)
**Controls**	**YES**	**YES**	**YES**	**YES**
**Regional Fixed**	**YES**	**YES**	**YES**	**YES**
**Year Fixed**	**YES**	**YES**	**YES**	**YES**
**Observations**	29997	38026	29955	37976
**Wald chi2**	5921.08	7979.09	2050.88	2764.38
**Pseudo R2**	0.3432	0.184	0.0237	0.0252

### 5.2 Heterogeneity analysis regression results

Heterogeneity Analysis by Age Groups: In line with established literature practices, this study categorizes individuals over 45 years into three age groups: 45–54 (middle-aged), 55–64 (low-aged), and over 65 (high-aged). This classification allows for a nuanced understanding of the effects of individual behaviors across different age cohorts. Table 7 reveals that for individuals under 65, physical activity significantly impacts self-assessed health, depression symptoms, and daily functioning. However, for those over 65, its influence is significant only on self-assessed health and daily functioning, with no notable effect on depression symptoms. Social activities demonstrate a significant positive impact on depression symptoms and daily functioning across all age groups and on self-rated health in the older cohort.

**Table 7:** Analysis of regression results based on age differences.

	Self-assessed Health	Depression symptoms	Daily Functioning
	(1)	(2)	(3)
**Physical Activity ×45~54**	-0.139***	-0.0831*	-0.284***
	(-3.31)	(-2.08)	(-5.39)
**Physical Activity ×55~64**	-0.158***	-0.0957*	-0.368***
	(-3.44)	(-2.21)	(-5.41)
**Physical Activity ×65above**	-0.132***	-0.000213	-0.330***
	(-3.33)	(-0.01)	(-6.15)
**Social Activity ×45~54**	-0.028	-0.129***	-0.181***
	(-0.85)	(-4.11)	(-4.09)
**Social Activity ×55~64**	-0.0343	-0.100**	-0.117*
	(-1.06)	(-3.22)	(-2.16)
**Social Activity ×65 above**	-0.103***	-0.129***	-0.190***
	(-3.45)	(-4.50)	(-4.30)
**Regional Fixed**	**YES**	**YES**	**YES**
**Year Fixed**	**YES**	**YES**	**YES**
**Observations**	**YES**	**YES**	**YES**
**Wald chi2**	14091	14031	9654
**Pseudo R2**	1104.61	1621.07	781.25
**Regional Fixed**	0.0268	0.0187	0.0679

Heterogeneity Analysis by Income: Reflecting current scholarly approaches, this study segregates individuals over 45 into income quintiles. Table 8 displays the regression outcomes of individual behaviors on self-assessed health, depression symptoms, and daily functioning across low-income, middle-income, and high-income groups. Physical activity markedly enhances self-rated health and daily functioning in low and lower-middle-income individuals, yet this effect diminishes among high-income elderly. Conversely, social activities substantially improve self-rated health, depression symptoms, and daily functioning in low- and middle-income groups, with the effect remaining insignificant in high-income individuals.

**Table 8:** Analysis of regression results based on income differences.

	Self-assessed Health	Depression symptoms	Daily Functioning
	(1)	(2)	(3)
Physical Activity * lower income	-0.171***	-0.0211	-0.319***
	(-3.53)	(-0.45)	(-5.15)
Physical Activity * Medium income	-0.129**	-0.0532	-0.354***
	(-2.91)	(-1.27)	(-5.60)
Physical Activity * high income	-0.0789	-0.0704	0.373
	(-0.61)	(-0.50)	(1.52)
Social Activity * lower income	-0.0781*	-0.154***	-0.195***
	(-2.13)	(-4.30)	(-3.91)
Social Activity * Medium income	-0.0398	-0.101**	-0.133*
	(-1.10)	(-2.87)	(-2.38)
Social Activity * high income	0.00838	0.00503	-0.354
	(-0.10)	(-0.94)	(-1.71)
**Regional Fixed**	**YES**	**YES**	**YES**
**Year Fixed**	**YES**	**YES**	**YES**
**Observations**	**YES**	**YES**	**YES**
**Wald chi2**	14091	14031	9654
**Pseudo R2**	1092.14	1607.9	784.61
**Regional Fixed**	0.0262	0.0184	0.0688

## 6. Conclusions and discussions

### 6.1 Conclusions

This paper, utilizing large-scale micro-survey data from CHARLS, employs ordered polynomial choice probit models to investigate the influence of preventive behaviors on self-assessed health, depressive symptoms, and daily functioning among middle-aged and older adults. The research reveals that such behaviors markedly enhance self-rated health and daily functioning, notably in combating depression symptoms, with social activities playing a particularly vital role for this demographic. Additionally, demographic and socio-economic factors, including gender, age, and education level, are found to significantly affect individuals’ self-rated health, depressive tendencies, and functional capabilities. The study underscores that primary preventive actions, such as engaging in physical and social activities, contribute to the health of middle-aged and older individuals by reducing the incidence of diseases. In contrast, secondary preventive behaviors, like regular health check-ups, positively impact health outcomes by facilitating early disease detection, enabling prompt medical intervention, and providing health alerts. Furthermore, heterogeneity analyses indicate that physical activities substantially boost self-rated health, alleviate depressive symptoms, and enhance daily functioning in those under 65 years of age, yet display no significant effect on depression symptoms in the over-65 cohort. Conversely, social activities considerably improve health, depression symptoms, and daily functioning in the over-65 age group. However, among higher-income individuals, the improvements in health, depression symptoms, and daily functioning attributed to social and physical activities are not as pronounced.

### 6.2 Discussions

The findings of this study clearly demonstrate significant age and income disparities in the impact of preventative behaviors on health outcomes, suggesting complex links between individual behaviors and socio-economic status. These findings are consistent with prior research, confirming the close connection between socio-economic status and preventative behaviors [66], as well as the causal relationships between individual behaviors such as physical and social activities and health outcomes [36,39,40]. Particularly, health check-up services, a crucial component of primary healthcare, exhibit complex interactions with health outcomes and other individual behaviors. Strategies to reduce the costs of health screenings or to enhance the accessibility of medical services are vital for encouraging preventative health check-ups. Ensuring that elderly individuals more broadly receive timely health checks can not only improve their health status but may also reduce overall medical expenditures. Additionally, socio-demographic variables such as gender, age, and educational level play a moderating role in the relationship between individual preventative behaviors and health outcomes. For example, the level of education can influence an individual’s ability to access and process health-related information, thereby indirectly affecting the adoption and effectiveness of preventative behaviors. These differences caused by socio-demographic characteristics necessitate considering the unique needs of different groups when devising health promotion strategies.

The findings of this study significantly inform current health promotion strategies and public health policy frameworks. It highlights the evolving need to address social needs for promoting healthy aging, beyond relying solely on older adults’ health awareness and initiative. This approach is vital in community health interventions. Offering more opportunities for physical activity and health screenings, especially to low-income or less educated elderly populations, could enhance their overall health. The research underscores the importance of considering factors like age, gender, education, and income in developing targeted health interventions, suggesting future research to explore the interplay of these factors further and the potential for social and policy interventions to enhance the benefits of preventive behaviors.

This study possesses several significant advantages. First, it utilized data from the China Health and Retirement Longitudinal Study (CHARLS), which, due to its extensive individual sample coverage, ensures that the results are highly representative of the middle-aged and elderly population in China. Second, by employing a panel ordered probit model, this research conducted a systematic and in-depth analysis of the impacts on various health outcomes. Additionally, the study incorporated multidimensional variables such as income and age into the model, allowing for a comprehensive assessment and explanation of the influencing factors.

However, there are some non-negligible limitations to this research. Firstly, the reliance on self-reported health data from participants may introduce reporting bias, affecting the objectivity and accuracy of the data. Secondly, although the study has attempted to control for a variety of potential confounding factors, it cannot guarantee the complete exclusion of all potential influencing variables. Lastly, given that the sample is limited to middle-aged and elderly individuals in China, the generalizability of the results may be limited in other countries or regions.

Despite these limitations, this study provides valuable insights into the relationship between preventative behaviors and health among the elderly in China, and it is of significant value in understanding the health behavior patterns of this population. However, considering cultural, socio-economic, and healthcare system differences, these results may not be universally applicable in other countries or regions. Therefore, future research should be conducted in different cultural and socio-economic contexts to validate these findings’ broad applicability and deepen our understanding of these relationships.
